# Breaking the Stereotype: Virulence Factor–Mediated Protection of Host Cells in Bacterial Pathogenesis

**DOI:** 10.1371/journal.ppat.1001057

**Published:** 2010-09-16

**Authors:** Stephanie R. Shames, B. Brett Finlay

**Affiliations:** Michael Smith Laboratories and Department of Microbiology and Immunology, University of British Columbia, Vancouver, British Columbia, Canada; The Fox Chase Cancer Center, United States of America

Bacterial pathogens have evolved extraordinary mechanisms to efficiently infect host organisms. A majority of these pathogens do so by delivering virulence factors into host cells, which act to dampen host defenses or utilize the host as a niche for replication. Although regulation of virulence factor expression by bacterial pathogens is a well known pathogenic mechanism [1], the concept of host-protective virulence factors is emerging. Recently, several strategies by which pathogens appear to be attenuating their own lethality towards host cells have been documented, suggesting that increased hostility and damage of host cells is not necessarily beneficial to the pathogen. Virulence is often defined as the ability of a pathogen to inflict damage on host cells, and the following discussion addresses the concept that increased virulence is not always beneficial to the pathogen, and moderating it to preserve host cells is a mechanism several pathogens use as part of their overall pathogenic strategy. This strategy is well known for obligate intracellular pathogens, but has become an emerging theme in extracellular and facultative intracellular bacteria.

*Yersinia* spp., *Shigella flexneri*, *Helicobacter pylori*, and diarrheagenic *Escherichia coli* are well known for their ability to kill host cells. For *Yersinia*, death of infected macrophages dampens cytokine release and enables the pathogen to propagate with minimal challenges from the immune system [2]. Two recent studies suggest that cytotoxicity caused by *Yersinia* species is tightly regulated. *Yersinia pestis*, the etiologic agent of plague, and gastroenteritis-inducing *Yersinia pseudotuberculosis* and *Yersinia enterocolitica* all encode a cytotoxic virulence factor called YopJ/P (YopJ in the two former species and YopP in the latter), which are translocated into infected cells via a type III secretion system (T3SS) [2], [3]. Altering the cytotoxicity of *Y. pseudotuberculosis* affects its virulence. Decreased secretion of YopJ was shown to enhance *Y. pseudotuberculosis* pathogenesis in vivo [4]. Similarly for *Y. pestis*, enhanced cytotoxicity results in decreased incidence of pneumonic plague in vivo [5]. Tight regulation of cytotoxicity by pathogenic *Yersinia* is an efficient virulence strategy. Increased apoptosis of infected immune cells decreases production of proinflammatory cytokines; however, some inflammation at the early stages of infection is thought to facilitate tissue damage necessary for movement of bacteria and infected cells to other sites of replication within the host [4].

**Figure 1:**
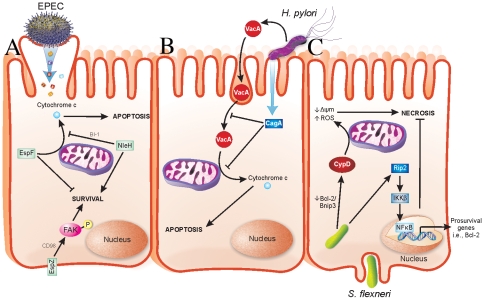
Strategies evolved by bacterial pathogens to restrain virulence. (A) EPEC injects effector proteins into intestinal epithelial cells (IECs) via a T3SS. EspF localizes to mitochondria and causes release of cytochrome *c* into the host cell cytosol, which results in apoptotic death of the host cells. NleH interacts with Bax inhibitor-1 (BI-1), which inhibits release of cytochrome *c* from mitochondria. EspZ interacts with CD98, which then stimulates phosphorylation of focal adhesion kinase (FAK) to promote survival. Localization of NleH and EspZ in host cells during early stages of EPEC infection is unclear and has been portrayed as shown for simplicity. (B) *H. pylori* injects virulence factors into gastric epithelial cells via a type IV secretion system in addition to secreting soluble toxins. VacA is an *H. pylori*–secreted toxin that enters cells by pinocytosis and penetrates intracellular endosome trafficking pathways. VacA causes release of cytochrome *c* from mitochondria of infected cells, thus mediating host cell apoptosis. CagA is a T4S virulence factor, which prevents both pinocytosis/trafficking and cytochrome *c* release by VacA. Functions of CagA are dependent on its phosphorylation state, not depicted here. (C) *S. flexneri* enters IECs from their basolateral surface and then resides in the cell cytoplasm. Prosurvival signaling is initiated by Nod1 activation of Rip2 signaling, which terminates in expression of pro-survival genes, including Bcl-2, via NFκB activation and nuclear translocation. Conversely, *S. flexneri* facilitates a decrease in the Bcl-2/Bnip3 ratio, which leads to CypD-mediated disruption of mitochondria and oxidative stress-induced necrotic cell death.

Enteropathogenic *Escherichia coli*, enterohaemorrhagic *E. coli* (EPEC and EHEC, respectively), and *Citrobacter rodentium* are attaching and effacing (A/E) pathogens that cause severe diarrheagenic disease [6]. The ability of A/E pathogens to kill intestinal epithelial cells has been well documented [7]–[14]. The type III secreted (T3S) effector EspF has a role in host cell death by causing mitochondrial-dependent apoptosis [11], [15]. We recently found that the T3S effector EspZ modulates cytotoxicity towards host cells. An EPEC *espZ* mutant (Δ*espZ*) caused enhanced cytotoxicity in host cells when compared to the wild-type strain [16], which was surprising since the Δ*espZ* strain is severely attenuated for virulence in vivo [17]. EspZ acts in part through the host transmembrane glycoprotein CD98 to activate focal adhesion kinase (FAK)-based survival pathways (Figure 1) [16]. Others found that the T3S effector NleH also dampens apoptosis of EPEC-infected cells, but via interaction with a Bcl-2-related protein involved in the mitochondrial death pathway (Figure 1) [18]. Unlike EspZ, NleH is not essential for EPEC colonization and only moderately impacts on A/E pathogen disease in vivo [19], [20]; however, there are likely other host-protective virulence factors that act redundantly to NleH during EPEC infection.

*H. pylori* causes apoptosis of infected gastric epithelial cells [21]. Apoptosis induction by *H. pylori* has been linked to a secreted toxin called VacA, which induces cytochrome *c* release from mitochondria (Figure 1) [22]. Recently, it was determined that VacA-mediated apoptosis is counteracted by a type IV secreted (T4S) protein called CagA by both blocking pinocytosis of VacA and inhibiting VacA-mediated cytochrome *c* release from mitochondria [23] (Figure 1). Interestingly, loss of CagA in a VacA^+^ *H. pylori* strain decreases bacterial colonization and the incidence of gastric hyperplasia, adenocarcinoma, and inflammation [24]. Similar to the aforementioned pathogens, *H. pylori* has evolved a delicate interplay between host-protective and -detrimental virulence factors that are able to fine-tune virulence while promoting their propagation.

*S. flexneri*, the etiologic agent of bacilliary dysentery, causes death of infected macrophages and epithelial cells [25]. Despite this, several host-protective strategies are employed by *S. flexneri*. The T3S effector OspE was recently found to enhance adhesion of infected host cells to the underlying extracellular matrix [26]. Whether OspE activates host cell survival pathways directly is unknown; however, its interaction with integrin-linked kinase inhibits sloughing of infected cells into the intestinal lumen [26], consequently preventing anoikis of *Shigella*-infected cells. An *ospE* mutant does not colonize as efficiently as wild-type *S. flexneri* in vivo; thus, OspE may enhance colonization by preventing premature release of infected cells [26]. Epithelial cells succumb to *S. flexneri* infection via necrotic cell death, which functions to release intracellular bacteria and enhance inflammation [25]. Interestingly, survival pathways involving Rip2/IKKβ/NFκB are activated early during infection, followed by mitochondrial dysfunction and necrotic cell death (Figure 1) [25]. The early expression of pro-survival genes may enable *S. flexneri* to postpone cell death in a similar manner to EPEC, thus ensuring greater bacterial load prior to dissemination. The mechanism(s) by which *S. flexneri* enhances NFκB-mediated pro-survival signals are unknown.

All of the above pathogens have evolved strategies to attenuate their own host-damaging virulence factors. In many of these scenarios, removal of host-protective mediators actually reduces pathogenicity of the bacteria. The observation that EPEC encodes a host-protective virulence factor that is essential for its pathogenesis suggests that protecting host cells may be a key to the pathogenic strategies of other bacterial pathogens. The concept of host-protective virulence factors is only just emerging, and we believe host-protective virulence factors will become more apparent in other pathogenic strategies and may become interesting targets to combat bacterial disease. Importantly, virulence phenotypes that appear counterintuitive should not be ignored. Future studies into pathogenic mechanisms of virulent bacteria will likely reveal important roles for effectors or regulatory mechanisms that help the host cell and promote bacterial pathogenesis.
